# (*E*,*Z*)-1-(4-Chloro­phen­yl)-5-phenyl-5-(phenyl­sulfan­yl)penta-2,4-dien-1-one

**DOI:** 10.1107/S160053681302312X

**Published:** 2013-08-31

**Authors:** Anna V. Vologzhanina, Dmitry M. Gusev, Alexander A. Golovanov, Valentina S. Pisareva

**Affiliations:** a Nesmeyanov Institute of Organoelement Compounds of the Russian Academy of Sciences, 119991 Moscow, Russian Federation; b Department of Chemical and Chemical Technology, Togliatti State University, 445667 Togliatti, Russian Federation

## Abstract

The penta-2,4-dien-1-one fragment of the title compound, C_23_H_17_ClOS, is twisted by 20.0 (3)°, as measured by the dihedral angle between the planes of the carbonyl group and its attached C atom and the distant C=C double bond and its attached C atom. The 4-chloro­phenyl group forms a dihedral angle of 4.0 (3)° with the adjacent carbonyl group. Conjugation between the phenyl ring and the C=C double bond is absent; the dihedral angle between the phenyl ring and the C—C=C fragment is 34.3 (2)°. In the crystal, mol­ecules are linked *via* C—H⋯O hydrogen bonds, forming chains parallel to the *b-*axis direction.

## Related literature

For the biological activity of chalcones, and their aryl­thio-containing derivatives, see: Chate *et al.* (2012 ▶); Nielsen *et al.* (2005 ▶); Wu *et al.* (2011 ▶), Karaman *et al.* (2012 ▶). For the synthesis and crystal structures of precursor 1,5-di­aryl­pent-2-en-4-yn-1-ones, see: Golovanov *et al.* (2013 ▶). For standard bond lengths, see: Allen *et al.* (1987 ▶).
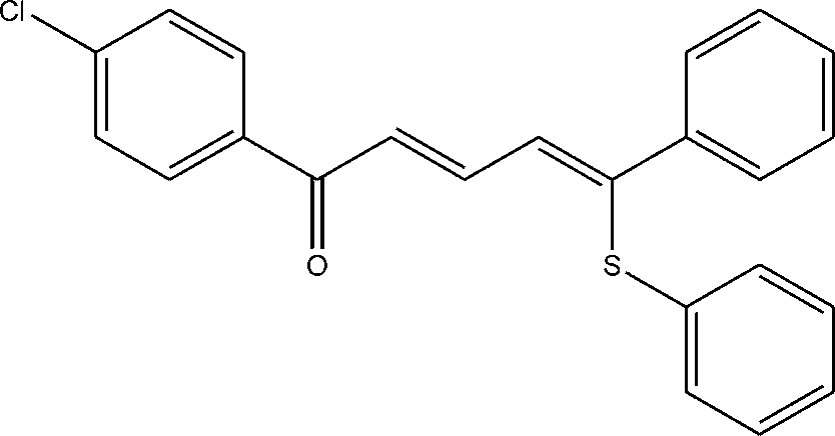


## Experimental

### 

#### Crystal data

C_23_H_17_ClOS
*M* *_r_* = 376.88
Orthorhombic, 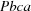

*a* = 8.2663 (11) Å
*b* = 11.1661 (13) Å
*c* = 39.478 (6) Å
*V* = 3643.9 (8) Å^3^
*Z* = 8
Mo *K*α radiation
μ = 0.33 mm^−1^
*T* = 120 K
0.38 × 0.08 × 0.07 mm

#### Data collection

Bruker APEXII CCD diffractometer
Absorption correction: multi-scan (*SADABS*; Sheldrick, 1998 ▶) *T*_min_ = 0.903, *T*_max_ = 0.967
20709 measured reflections
5311 independent reflections
3104 reflections with *I* > 2σ(*I*)
*R*_int_ = 0.088

#### Refinement

*R*[*F* ^2^ > 2σ(*F* ^2^)] = 0.049
*wR*(*F* ^2^) = 0.100
*S* = 1.00
5311 reflections
235 parameters
H-atom parameters constrained
Δρ_max_ = 0.38 e Å^−3^
Δρ_min_ = −0.37 e Å^−3^

### 

Data collection: *APEX2* (Bruker, 2005 ▶); cell refinement: *SAINT* (Bruker, 2005 ▶); data reduction: *SAINT*; program(s) used to solve structure: *SHELXTL* (Sheldrick, 2008 ▶); program(s) used to refine structure: *SHELXTL*; molecular graphics: *SHELXTL*; software used to prepare material for publication: *SHELXTL*.

## Supplementary Material

Crystal structure: contains datablock(s) I. DOI: 10.1107/S160053681302312X/ld2112sup1.cif

Structure factors: contains datablock(s) I. DOI: 10.1107/S160053681302312X/ld2112Isup2.hkl

Supplementary material file. DOI: 10.1107/S160053681302312X/ld2112Isup3.cml

Additional supplementary materials:  crystallographic information; 3D view; checkCIF report

## Figures and Tables

**Table 1:** Hydrogen-bond geometry (Å, °)

*D*—H⋯*A*	*D*—H	H⋯*A*	*D*⋯*A*	*D*—H⋯*A*
C7—H7*A*⋯O1^i^	0.95	2.57	3.515 (3)	178

Symmetry code: (i) 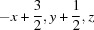.

**Fig. 1.**
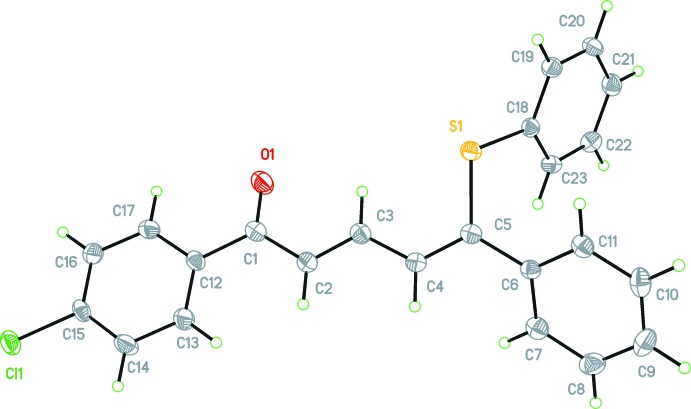
The molecular structure of the title compound. Displacement ellipsoids are drawn at 50% probability level.

**Fig. 2.**
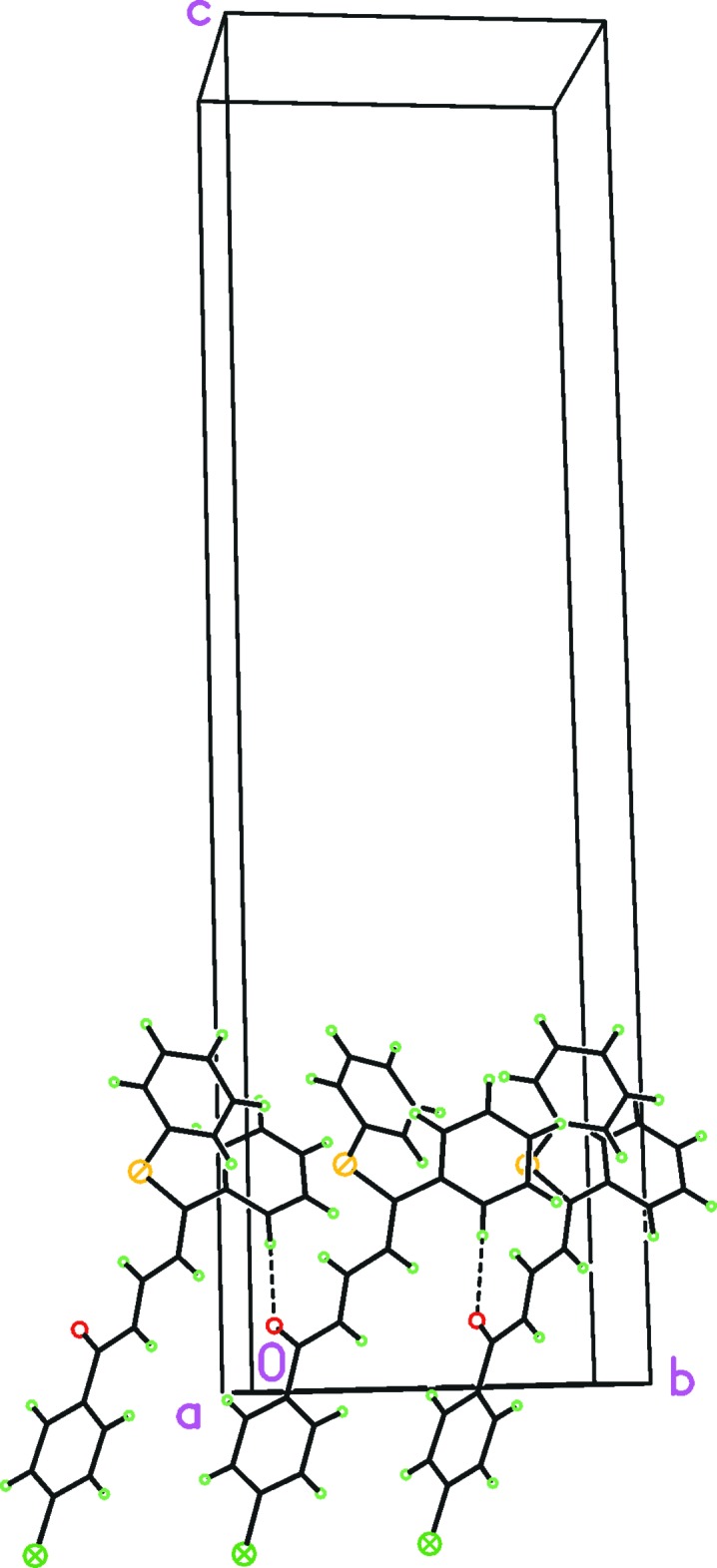
The C—H···O bonded chain viewed down the a axis. Dashed lines indicate hydrogen bonds.

## supplementary crystallographic information

### 1. Comment

The family of chalcones exhibit antibiotic (Nielsen *et al.*, 2005) and anti-inflammatory (Wu *et al.*, 2011) activity. Arylthio-containing ketones are also active against some human pathogenic microorganisms (Chate *et al.*, 2012; Karaman *et al.*, 2012). Thus, a molecule which contains both fragments may have a high biological effect. Herein, we present the structure of (E, *Z*)-1-(4-chlorophenyl)-5-phenyl-5-phenylthio-penta-2,4-dien-1-one prepared by Michael-type addition reaction between thiophenol and 1-(4-chlorophenyl)-5-phenyl-2-penten-4-yn-1-one.

All bond lengths have characteristic values (Allen *et al.*, 1987), although the length of the C3—C4 bond (1.429 (3) Å) indicates some electron delocalization along polyene C═C—C═C chain. The S—C distances of 1.769 (2) and 1.774 (2) Å, are slightly shortened due to mesomeric effect of sulfur electron pairs. The penta-2,4-dien-1-one fragment is twisted, the angle between two meanplanes (O1═C1—C2 and C3—C4═C5) is equal to 20.0 (3) °. The 4-chlorophenyl ring makes with the carbonyl group a dihedral angle of 4.0 (3) °. A dihedral angle between the phenyl ring and C3—C4═C5 fragment is 34.3 (2)°.

The molecules are linked in the crystal *via* C7—H7A···O bonds into chains parallel to the crystallographic *b* axis. It is worth mentioning that the C—H···O bonds which involve the hydrogen atom at *o* position of phenyl ring are typical for 1,5-diarylsubstituted penten-yn-ones (Golovanov, *et al.*, 2013).

### 2. Experimental

Three drops of triethylamine were added to a solution of 1-(4-chlorophenyl)-5-phenylpent-2-en-4-yn-1-one (322 mg, 1.21 mmol) and thiophenol (133 mg, 1.21 mmol) in 3 ml 95% ethanol. After 12 h, the precipitated yellow crystals were filtered and washed with 2 ml of cold 40% alcohol. Yield 82%. The single crystal was obtained from mixture of acetone and water. M.p. 366–367K.

### 3. Refinement

All non-H atoms were refined anisotropically. Hydrogen atoms were positioned geometrically and refined isotropically being constrained to ride on their adjacent carbon atoms with *U*_iso_(H) = 1.2*U*_eq_(C).

### Crystal data

**Table:**

C_23_H_17_ClOS	*D*_x_ = 1.374 Mg m^−^^3^
*M**_r_* = 376.88	Melting point = 366–280 K
Orthorhombic, *P**b**c**a*	Mo *K*α radiation, λ = 0.71073 Å
Hall symbol: -P 2ac 2ab	Cell parameters from 1957 reflections
*a* = 8.2663 (11) Å	θ = 2.7–27.8°
*b* = 11.1661 (13) Å	µ = 0.33 mm^−^^1^
*c* = 39.478 (6) Å	*T* = 120 K
*V* = 3643.9 (8) Å^3^	Needle, yellow
*Z* = 8	0.38 × 0.08 × 0.07 mm
*F*(000) = 1568	

### Data collection

**Table:**

Bruker APEXII CCD diffractometer	5311 independent reflections
Radiation source: fine-focus sealed tube	3104 reflections with *I* > 2σ(*I*)
Graphite monochromator	*R*_int_ = 0.088
ω scans	θ_max_ = 30.0°, θ_min_ = 2.1°
Absorption correction: multi-scan (*SADABS*; Sheldrick, 1998)	*h* = −11→11
*T*_min_ = 0.903, *T*_max_ = 0.967	*k* = −11→15
20709 measured reflections	*l* = −55→38

### Refinement

**Table:**

Refinement on *F*^2^	Primary atom site location: structure-invariant direct methods
Least-squares matrix: full	Secondary atom site location: difference Fourier map
*R*[*F*^2^ > 2σ(*F*^2^)] = 0.049	Hydrogen site location: inferred from neighbouring sites
*wR*(*F*^2^) = 0.100	H-atom parameters constrained
*S* = 1.00	*w* = 1/[σ^2^(*F*_o_^2^) + (0.019*P*)^2^ + 2.8*P*] where *P* = (*F*_o_^2^ + 2*F*_c_^2^)/3
5311 reflections	(Δ/σ)_max_ < 0.001
235 parameters	Δρ_max_ = 0.38 e Å^−^^3^
0 restraints	Δρ_min_ = −0.37 e Å^−^^3^

### Special details

**Table:**

Experimental. IR (KBr), ν/cm^-1^: 3051, 1648, 1589, 1573, 1559, 1481, 1441, 1397, 1356, 1333, 1272, 1225, 1176, 1091, 1025, 1009, 939. ^1^H NMR (400 MHz, CDCl_3_): δ = 7.02 (d, 1H, J = 11.2 Hz), 7.12 (d, 1H, J = 14.9 Hz), 7.20–8.00 (m, 14H), 8.27 (dd, 1H, J = 11.2 Hz, J = 15.0 Hz). ^13^C NMR (100 MHz, CDCl3): 77.5, 123.2, 127.3, 129.0, 130.1, 132.3, 134.7, 136.6, 139.2, 141.5, 153.9, 189.5. Anal. Calcd. for C_23_H_17_ClSO: C, 73.29; H, 4.67. Found: C, 73.33; H, 4.56.
Geometry. All e.s.d.'s (except the e.s.d. in the dihedral angle between two l.s. planes) are estimated using the full covariance matrix. The cell e.s.d.'s are taken into account individually in the estimation of e.s.d.'s in distances, angles and torsion angles; correlations between e.s.d.'s in cell parameters are only used when they are defined by crystal symmetry. An approximate (isotropic) treatment of cell e.s.d.'s is used for estimating e.s.d.'s involving l.s. planes.
Refinement. Refinement of *F*^2^ against ALL reflections. The weighted *R*-factor *wR* and goodness of fit *S* are based on *F*^2^, conventional *R*-factors *R* are based on *F*, with *F* set to zero for negative *F*^2^. The threshold expression of *F*^2^ > σ(*F*^2^) is used only for calculating *R*-factors(gt) *etc*. and is not relevant to the choice of reflections for refinement. *R*-factors based on *F*^2^ are statistically about twice as large as those based on *F*, and *R*- factors based on ALL data will be even larger.

### Fractional atomic coordinates and isotropic or equivalent isotropic displacement parameters (Å^2^)

**Table:**

	*x*	*y*	*z*	*U*_iso_*/*U*_eq_	
S1	0.88212 (7)	0.25480 (5)	0.159299 (14)	0.02170 (13)	
Cl1	0.91964 (8)	−0.02110 (5)	−0.110391 (14)	0.02792 (15)	
O1	0.6697 (2)	0.08592 (16)	0.04724 (4)	0.0328 (4)	
C1	0.7800 (3)	0.1369 (2)	0.03200 (6)	0.0222 (5)	
C2	0.8736 (3)	0.2331 (2)	0.04818 (6)	0.0220 (5)	
H2A	0.9446	0.2803	0.0348	0.026*	
C3	0.8611 (3)	0.2558 (2)	0.08142 (5)	0.0210 (5)	
H3A	0.7946	0.2043	0.0946	0.025*	
C4	0.9409 (3)	0.35209 (19)	0.09840 (6)	0.0207 (5)	
H4A	0.9973	0.4078	0.0845	0.025*	
C5	0.9444 (3)	0.37216 (19)	0.13219 (6)	0.0188 (5)	
C6	1.0132 (3)	0.48280 (19)	0.14683 (6)	0.0173 (5)	
C7	1.0076 (3)	0.5896 (2)	0.12817 (6)	0.0233 (5)	
H7A	0.9575	0.5906	0.1065	0.028*	
C8	1.0748 (3)	0.6937 (2)	0.14119 (7)	0.0294 (6)	
H8A	1.0716	0.7655	0.1283	0.035*	
C9	1.1464 (3)	0.6935 (2)	0.17291 (7)	0.0293 (6)	
H9A	1.1925	0.7648	0.1817	0.035*	
C10	1.1504 (3)	0.5892 (2)	0.19162 (6)	0.0266 (6)	
H10A	1.1986	0.5890	0.2135	0.032*	
C11	1.0847 (3)	0.4850 (2)	0.17873 (6)	0.0213 (5)	
H11A	1.0884	0.4137	0.1918	0.026*	
C12	0.8177 (3)	0.1007 (2)	−0.00365 (6)	0.0199 (5)	
C13	0.9341 (3)	0.1579 (2)	−0.02328 (6)	0.0275 (6)	
H13A	0.9929	0.2234	−0.0141	0.033*	
C14	0.9652 (3)	0.1205 (2)	−0.05610 (6)	0.0292 (6)	
H14A	1.0441	0.1605	−0.0695	0.035*	
C15	0.8809 (3)	0.0248 (2)	−0.06919 (5)	0.0206 (5)	
C16	0.7655 (3)	−0.0342 (2)	−0.05040 (6)	0.0262 (6)	
H16A	0.7082	−0.1002	−0.0597	0.031*	
C17	0.7343 (3)	0.0043 (2)	−0.01770 (6)	0.0261 (5)	
H17A	0.6546	−0.0358	−0.0046	0.031*	
C18	0.7446 (3)	0.32191 (19)	0.18812 (6)	0.0176 (5)	
C19	0.7105 (3)	0.2586 (2)	0.21755 (6)	0.0209 (5)	
H19A	0.7657	0.1857	0.2222	0.025*	
C20	0.5960 (3)	0.3016 (2)	0.24015 (6)	0.0229 (5)	
H20A	0.5721	0.2575	0.2601	0.028*	
C21	0.5163 (3)	0.4082 (2)	0.23384 (6)	0.0243 (5)	
H21A	0.4386	0.4380	0.2495	0.029*	
C22	0.5510 (3)	0.4712 (2)	0.20447 (6)	0.0232 (5)	
H22A	0.4961	0.5442	0.2000	0.028*	
C23	0.6647 (3)	0.4293 (2)	0.18159 (6)	0.0204 (5)	
H23A	0.6879	0.4735	0.1616	0.024*	

### Atomic displacement parameters (Å^2^)

**Table:**

	*U*^11^	*U*^22^	*U*^33^	*U*^12^	*U*^13^	*U*^23^
S1	0.0278 (3)	0.0179 (3)	0.0194 (3)	0.0019 (3)	0.0046 (3)	0.0014 (2)
Cl1	0.0348 (3)	0.0317 (3)	0.0173 (3)	0.0022 (3)	0.0017 (3)	−0.0028 (2)
O1	0.0357 (11)	0.0387 (10)	0.0240 (9)	−0.0100 (9)	0.0075 (8)	−0.0041 (8)
C1	0.0218 (13)	0.0246 (13)	0.0202 (13)	0.0011 (10)	0.0013 (10)	0.0018 (10)
C2	0.0254 (12)	0.0218 (12)	0.0188 (11)	−0.0005 (10)	−0.0001 (10)	0.0009 (9)
C3	0.0217 (12)	0.0217 (11)	0.0197 (11)	0.0028 (10)	0.0003 (9)	0.0012 (10)
C4	0.0218 (12)	0.0204 (12)	0.0198 (12)	−0.0018 (10)	0.0022 (10)	0.0011 (9)
C5	0.0176 (11)	0.0188 (11)	0.0200 (11)	0.0027 (9)	0.0012 (10)	0.0020 (9)
C6	0.0152 (11)	0.0184 (11)	0.0183 (11)	0.0014 (9)	0.0032 (9)	0.0004 (9)
C7	0.0226 (13)	0.0239 (12)	0.0233 (13)	0.0047 (10)	0.0025 (10)	0.0005 (10)
C8	0.0338 (15)	0.0199 (13)	0.0344 (15)	0.0018 (11)	0.0083 (12)	0.0022 (11)
C9	0.0247 (14)	0.0253 (13)	0.0381 (15)	−0.0041 (11)	0.0018 (12)	−0.0099 (12)
C10	0.0208 (13)	0.0343 (14)	0.0247 (13)	0.0020 (11)	−0.0023 (11)	−0.0093 (11)
C11	0.0204 (12)	0.0230 (12)	0.0205 (11)	0.0041 (10)	0.0020 (10)	−0.0001 (10)
C12	0.0230 (12)	0.0217 (12)	0.0149 (11)	0.0028 (10)	−0.0004 (10)	0.0012 (9)
C13	0.0326 (15)	0.0299 (14)	0.0199 (12)	−0.0100 (12)	0.0000 (11)	−0.0037 (10)
C14	0.0281 (14)	0.0381 (15)	0.0213 (13)	−0.0121 (12)	0.0055 (11)	−0.0006 (11)
C15	0.0244 (12)	0.0252 (12)	0.0122 (10)	0.0059 (11)	−0.0013 (9)	−0.0003 (9)
C16	0.0330 (14)	0.0240 (13)	0.0216 (13)	−0.0065 (11)	−0.0009 (11)	−0.0022 (10)
C17	0.0297 (14)	0.0263 (13)	0.0223 (12)	−0.0086 (11)	0.0043 (11)	0.0000 (11)
C18	0.0171 (11)	0.0189 (11)	0.0168 (11)	−0.0018 (9)	−0.0001 (9)	−0.0013 (9)
C19	0.0228 (12)	0.0198 (12)	0.0203 (12)	0.0004 (10)	−0.0027 (9)	0.0016 (10)
C20	0.0266 (13)	0.0259 (12)	0.0164 (11)	−0.0048 (11)	0.0009 (10)	0.0033 (9)
C21	0.0222 (13)	0.0273 (13)	0.0232 (13)	−0.0016 (10)	0.0045 (10)	−0.0045 (10)
C22	0.0215 (13)	0.0212 (12)	0.0271 (13)	0.0000 (10)	0.0003 (10)	−0.0003 (10)
C23	0.0211 (12)	0.0198 (11)	0.0202 (12)	−0.0018 (10)	0.0012 (10)	0.0030 (9)

### Geometric parameters (Å, º)

**Table:**

S1—C5	1.769 (2)	C11—H11A	0.9500
S1—C18	1.774 (2)	C12—C13	1.391 (3)
Cl1—C15	1.735 (2)	C12—C17	1.393 (3)
O1—C1	1.231 (3)	C13—C14	1.386 (3)
C1—C2	1.470 (3)	C13—H13A	0.9500
C1—C12	1.497 (3)	C14—C15	1.377 (3)
C2—C3	1.340 (3)	C14—H14A	0.9500
C2—H2A	0.9500	C15—C16	1.376 (3)
C3—C4	1.429 (3)	C16—C17	1.385 (3)
C3—H3A	0.9500	C16—H16A	0.9500
C4—C5	1.353 (3)	C17—H17A	0.9500
C4—H4A	0.9500	C18—C19	1.389 (3)
C5—C6	1.478 (3)	C18—C23	1.394 (3)
C6—C11	1.391 (3)	C19—C20	1.387 (3)
C6—C7	1.402 (3)	C19—H19A	0.9500
C7—C8	1.388 (3)	C20—C21	1.384 (3)
C7—H7A	0.9500	C20—H20A	0.9500
C8—C9	1.385 (3)	C21—C22	1.386 (3)
C8—H8A	0.9500	C21—H21A	0.9500
C9—C10	1.379 (3)	C22—C23	1.384 (3)
C9—H9A	0.9500	C22—H22A	0.9500
C10—C11	1.381 (3)	C23—H23A	0.9500
C10—H10A	0.9500		
			
C5—S1—C18	105.17 (10)	C13—C12—C1	122.9 (2)
O1—C1—C2	121.0 (2)	C17—C12—C1	118.7 (2)
O1—C1—C12	119.2 (2)	C14—C13—C12	120.7 (2)
C2—C1—C12	119.8 (2)	C14—C13—H13A	119.6
C3—C2—C1	121.5 (2)	C12—C13—H13A	119.6
C3—C2—H2A	119.2	C15—C14—C13	119.4 (2)
C1—C2—H2A	119.2	C15—C14—H14A	120.3
C2—C3—C4	124.5 (2)	C13—C14—H14A	120.3
C2—C3—H3A	117.8	C16—C15—C14	121.3 (2)
C4—C3—H3A	117.8	C16—C15—Cl1	119.48 (18)
C5—C4—C3	126.7 (2)	C14—C15—Cl1	119.17 (18)
C5—C4—H4A	116.7	C15—C16—C17	118.9 (2)
C3—C4—H4A	116.7	C15—C16—H16A	120.6
C4—C5—C6	122.2 (2)	C17—C16—H16A	120.6
C4—C5—S1	117.89 (17)	C16—C17—C12	121.3 (2)
C6—C5—S1	119.65 (17)	C16—C17—H17A	119.4
C11—C6—C7	118.3 (2)	C12—C17—H17A	119.4
C11—C6—C5	122.2 (2)	C19—C18—C23	119.8 (2)
C7—C6—C5	119.5 (2)	C19—C18—S1	116.83 (17)
C8—C7—C6	120.3 (2)	C23—C18—S1	123.27 (17)
C8—C7—H7A	119.8	C20—C19—C18	120.0 (2)
C6—C7—H7A	119.8	C20—C19—H19A	120.0
C9—C8—C7	120.3 (2)	C18—C19—H19A	120.0
C9—C8—H8A	119.9	C21—C20—C19	120.5 (2)
C7—C8—H8A	119.9	C21—C20—H20A	119.8
C10—C9—C8	119.8 (2)	C19—C20—H20A	119.8
C10—C9—H9A	120.1	C20—C21—C22	119.2 (2)
C8—C9—H9A	120.1	C20—C21—H21A	120.4
C9—C10—C11	120.3 (2)	C22—C21—H21A	120.4
C9—C10—H10A	119.9	C23—C22—C21	121.0 (2)
C11—C10—H10A	119.9	C23—C22—H22A	119.5
C10—C11—C6	121.1 (2)	C21—C22—H22A	119.5
C10—C11—H11A	119.5	C22—C23—C18	119.5 (2)
C6—C11—H11A	119.5	C22—C23—H23A	120.3
C13—C12—C17	118.4 (2)	C18—C23—H23A	120.3
			
O1—C1—C2—C3	11.0 (4)	O1—C1—C12—C17	−4.6 (3)
C12—C1—C2—C3	−169.6 (2)	C2—C1—C12—C17	176.0 (2)
C1—C2—C3—C4	−176.1 (2)	C17—C12—C13—C14	0.6 (4)
C2—C3—C4—C5	−173.4 (2)	C1—C12—C13—C14	179.6 (2)
C3—C4—C5—C6	−172.1 (2)	C12—C13—C14—C15	−0.7 (4)
C3—C4—C5—S1	14.1 (3)	C13—C14—C15—C16	0.4 (4)
C18—S1—C5—C4	−131.58 (19)	C13—C14—C15—Cl1	179.6 (2)
C18—S1—C5—C6	54.5 (2)	C14—C15—C16—C17	0.0 (4)
C4—C5—C6—C11	−150.1 (2)	Cl1—C15—C16—C17	−179.20 (19)
S1—C5—C6—C11	23.6 (3)	C15—C16—C17—C12	−0.2 (4)
C4—C5—C6—C7	29.7 (3)	C13—C12—C17—C16	−0.1 (4)
S1—C5—C6—C7	−156.70 (18)	C1—C12—C17—C16	−179.2 (2)
C11—C6—C7—C8	1.2 (3)	C5—S1—C18—C19	−163.19 (18)
C5—C6—C7—C8	−178.6 (2)	C5—S1—C18—C23	20.6 (2)
C6—C7—C8—C9	−0.7 (4)	C23—C18—C19—C20	0.7 (3)
C7—C8—C9—C10	−0.1 (4)	S1—C18—C19—C20	−175.65 (18)
C8—C9—C10—C11	0.6 (4)	C18—C19—C20—C21	−0.8 (3)
C9—C10—C11—C6	−0.1 (4)	C19—C20—C21—C22	0.6 (4)
C7—C6—C11—C10	−0.8 (3)	C20—C21—C22—C23	−0.4 (4)
C5—C6—C11—C10	179.0 (2)	C21—C22—C23—C18	0.3 (4)
O1—C1—C12—C13	176.4 (2)	C19—C18—C23—C22	−0.5 (3)
C2—C1—C12—C13	−3.0 (3)	S1—C18—C23—C22	175.62 (18)

### Hydrogen-bond geometry (Å, º)

**Table:**

*D*—H···*A*	*D*—H	H···*A*	*D*···*A*	*D*—H···*A*
C7—H7*A*···O1^i^	0.95	2.57	3.515 (3)	178

Symmetry code: (i) −*x*+3/2, *y*+1/2, *z*.
